# Exploring AI’s Potential in Papilledema Diagnosis to Support Dermatological Treatment Decisions in Rural Healthcare

**DOI:** 10.3390/diagnostics15192547

**Published:** 2025-10-09

**Authors:** Jonathan Shapiro, Mor Atlas, Naomi Fridman, Itay Cohen, Ziad Khamaysi, Mahdi Awwad, Naomi Silverstein, Tom Kozlovsky, Idit Maharshak

**Affiliations:** 1 Maccabi Healthcare Services, Tel Aviv 6817110, Israel; 2 Business Administration Faculty, Ono Academic College, Kiryat Ono 5510701, Israel; atlasm@gmail.com; 3 NF Algorithms & AI, Tel Aviv 6248906, Israel; naomi.fridman@gmail.com; 4 Rutgers School of Public Health, Rutgers University, Piscataway, NJ 08854, USA; itay3012@gmail.com; 5 Department of Dermatology, Rambam Health Care Campus, Haifa 3109601, Israel; zkhamaysi@gmail.com; 6 The Bruce and Ruth Rappaport Faculty of Medicine, Technion—Israel Institute of Technology, Haifa 3525433, Israel; 7 Tzafon Medical Center, Ophthalmology Unit, Tiberias 1521000, Israel; mhdywd375@gmail.com; 8 Technion Institute of Technology, Haifa 3200003, Israel; naomish@campus.technion.ac.il; 9 Department of Ophthalmology, Edith Wolfson Medical Center, Holon 5822012, Israel; tomkozlovsky@gmail.com (T.K.); imaharshak@gmail.com (I.M.); 10 Gray School of Medicine, Tel-Aviv University, Tel Aviv 6997801, Israel

**Keywords:** dermatology, papilledema, artificial intelligence, ChatGPT, fundus photography, GPT-4o, ResNet CNN, ophthalmology, med-induced ICP, accuracy

## Abstract

**Background**: Papilledema, an ophthalmic finding associated with increased intracranial pressure, is often induced by dermatological medications, including corticosteroids, isotretinoin, and tetracyclines. Early detection is crucial for preventing irreversible optic nerve damage, but access to ophthalmologic expertise is often limited in rural settings. Artificial intelligence (AI) may enable the automated and accurate detection of papilledema from fundus images, thereby supporting timely diagnosis and management. **Objective**: The primary objective of this study was to explore the diagnostic capability of ChatGPT-4o, a general large language model with multimodal input, in identifying papilledema from fundus photographs. For context, its performance was compared with a ResNet-based convolutional neural network (CNN) specifically fine-tuned for ophthalmic imaging, as well as with the assessments of two human ophthalmologists. The focus was on applications relevant to dermatological care in resource-limited environments. **Methods**: A dataset of 1094 fundus images (295 papilledema, 799 normal) was preprocessed and partitioned into a training set and a test set. The ResNet model was fine-tuned using discriminative learning rates and a one-cycle learning rate policy. GPT-4o and two human evaluators (a senior ophthalmologist and an ophthalmology resident) independently assessed the test images. Diagnostic metrics including sensitivity, specificity, positive predictive value (PPV), negative predictive value (NPV), accuracy, and Cohen’s Kappa, were calculated for each evaluator. **Results**: GPT-4o, when applied to papilledema detection, achieved an overall accuracy of 85.9% with substantial agreement beyond chance (Cohen’s Kappa = 0.72), but lower specificity (78.9%) and positive predictive value (73.7%) compared to benchmark models. For context, the ResNet model, fine-tuned for ophthalmic imaging, reached near-perfect accuracy (99.5%, Kappa = 0.99), while two human ophthalmologists achieved accuracies of 96.0% (Kappa ≈ 0.92). **Conclusions**: This study explored the capability of GPT-4o, a large language model with multimodal input, for detecting papilledema from fundus photographs. GPT-4o achieved moderate diagnostic accuracy and substantial agreement with the ground truth, but it underperformed compared to both a domain-specific ResNet model and human ophthalmologists. These findings underscore the distinction between generalist large language models and specialized diagnostic AI: while GPT-4o is not optimized for ophthalmic imaging, its accessibility, adaptability, and rapid evolution highlight its potential as a future adjunct in clinical screening, particularly in underserved settings. These findings also underscore the need for validation on external datasets and real-world clinical environments before such tools can be broadly implemented.

## 1. Introduction

Papilledema is a condition characterized by swelling of the optic disks due to increased intracranial pressure (ICP). Ophthalmologists consider papilledema an emergency clinical finding and, thus, urgently refer such patients for further evaluation and imaging in the ER to rule out a space-occupying lesion in the brain and to prevent permanent damage to the compressed optic nerves [1]. Papilledema is traditionally associated with neurological or systemic disorders but can also be induced by medications used in dermatology. Withdrawal from prolonged systemic corticosteroid treatment, vitamin A derivatives, i.e., isotretinoin, and tetracyclines, are associated with intracranial hypertension, thereby causing optic disk edema [2,3,4,5]. This presents a diagnostic challenge, particularly in rural areas with limited access to specialized ophthalmic evaluation.

Corticosteroids have a useful role as anti-inflammatory and immunosuppressant agents for treating an array of dermatological conditions [6]. However, long-term therapy and abrupt discontinuation of corticosteroids are associated with various neurologic complications, including intracranial hypertension (IH). They may alter CSF dynamics and possibly increase ICP, ultimately creating papilledema [3,7]. Vitamin A derivatives, such as isotretinoin, are commonly prescribed for severe acne and other keratinization disorders. Although isotretinoin is highly effective for treating severe acne, it can induce IH, particularly if used in high doses and when combined with tetracyclines. It is postulated that isotretinoin may act on CSF production or reabsorption, causing a rise in ICP [8,9]. A physician reporting system identified 181 cases of intracranial hypertension linked to isotretinoin, with symptoms appearing on average 2.3 months after exposure. 24% of patients had taken tetracycline around the same time. Six patients experienced recurrent symptoms when re-challenged with isotretinoin after discontinuation [10].

Tetracyclines, including doxycycline and minocycline, are broad-spectrum antibiotic drugs frequently prescribed to treat acne and rosacea due to their antibacterial and anti-inflammatory properties. However, these agents may act as a predisposing factor for IH, particularly among young women, in the age group that is frequently treated with them. Although the underlying mechanism remains unclear, it is thought to involve alterations in cerebrospinal fluid dynamics or vascular effects [2,11,12,13].

Artificial intelligence (AI) revolutionizes ophthalmic practice by enhancing the identification, recognition, and management of eye diseases. AI platforms, primarily through deep learning algorithms, are reaching a new level of screening and diagnosis of ocular diseases like diabetic retinopathy, age-related macular degeneration, and glaucoma, achieving sensitivities and specificities of over 90% [14,15]. Such systems detect subtle pathological changes in retinal images, allowing for early detection that supports large-scale screening activities, especially in underserved areas [16,17]. In the management of glaucoma, AI employs optical coherence tomography (OCT) data to predict disease progression and future functional losses, which enables timely interventions [18]. AI is transforming ophthalmology by enhancing diagnostic accuracy, enabling personalized treatment, and addressing care delivery gaps. These innovations are expanding access to high-quality care, reducing disparities, and improving patient outcomes globally [19,20].

Recent advances in AI and machine learning are reshaping clinicians’ approaches to diagnosing and managing complex medical conditions, such as papilledema, primarily through the use of tools like ChatGPT to analyze and interpret clinical data, including fundoscopic images, for preliminary assessment and decision support [20,21]. By importing the appropriate high-definition fundoscopic images and clinical backgrounds into an AI model, a clinician using AI can detect critical findings such as swelling of the optic disk, hemorrhages, or obliteration of the optic cup, which are typical indicators of papilledema [22]. When provided with relevant, specific patient data, such as medical history and medication use, ChatGPT can generate a differential diagnosis while providing further investigation directions [23].

For example, a recent study revealed that a specialized deep learning system (DLS) can reliably differentiate between optic disk drusen (ODD) and papilledema, even in cases of buried ODD and mild-to-moderate papilledema (Sensitivity: 78.4% (95% CI, 72.2–84.7%), Specificity: 91.3% (95% CI, 87.0–96.4%)) [24]. Moreover, the U-Net deep learning model, the first automated system for clinical detection and grading of papilledema, achieved outstanding performance with 99.82% sensitivity, 98.65% specificity, and 99.89% accuracy [25]. Another large study validated a deep-learning system for detecting papilledema using 14,341 fundus images, achieving high accuracy (AUC 0.99), sensitivity (96.4%), and specificity (84.7%), highlighting its potential for automated diagnosis [26]. Relatively, ChatGPT-4o demonstrated superior diagnostic accuracy over Gemini Advanced, correctly identifying 52% vs. 30% of surgical retina cases and 78% vs. 43% of medical retina cases, while Gemini Advanced failed to recognize OCTA scans without structural images, mistaking them for artwork, highlighting ChatGPT-4o’s advantage despite its limited diagnostic range [27].

Studies show the rising benefits of artificial intelligence in aiding ophthalmological and dermatological practice. In ophthalmology, for instance, AI algorithms can detect papilledema and other optic nerve pathologies using fundoscopic images, showing promising sensitivities and specificities in supporting early diagnosis and timely referrals [28,29]. Similarly, in dermatology, AI has been successfully applied to identifying skin lesions and determining systemic associations [30,31,32]. The platform’s ability to obtain visual and dermatological data analysis further facilitates its multidisciplinary management. The integration of clinical expertise with AI-based diagnostic tools enhances diagnostic accuracy, streamlines clinical workflows, and enables more effective management of papilledema. This combination thus both improves patient outcomes and eases the load on healthcare systems, highlighting the transformative potential of AI in modern medicine.

A significant potential application of artificial intelligence (AI) in medicine is its ability to enhance diagnostic capabilities in resource-limited settings. One such scenario involves dermatological patients receiving isotretinoin treatment who may present with headache symptoms. In rural or underserved areas where access to ophthalmologists may be limited, AI-based tools capable of analyzing fundus images may serve as critical diagnostic aids. The availability of such AI-driven tools could enable healthcare providers, such as dermatologists or general practitioners, to quickly identify signs of papilledema and make informed decisions about whether further specialized evaluation is needed. By improving early detection of papilledema, AI technology can enhance patient safety and optimize the management of patients treated with isotretinoin in remote settings. The primary focus of this study was to explore the diagnostic capability of GPT-4o, a large language model with multimodal input, for detecting papilledema. For benchmarking, its performance was evaluated against a ResNet-based convolutional neural network (CNN) fine-tuned for ophthalmic imaging, as well as against the assessments of two human ophthalmologists.

## 2. Methods

**Figure 1:**
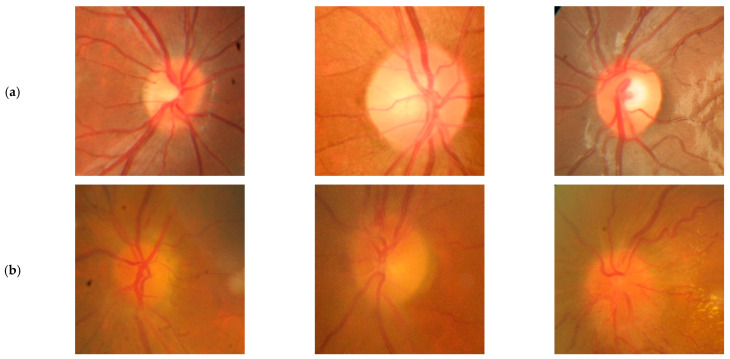
Representative fundus images of normal optic disks and optic disks with papilledema, used for evaluation. (**a**) Normal optic disk (**b**) Papilledema. images were taken from Awesome-Medical-Dataset/resources/Papilledema.md at main·openmedlab/Awesome-Medical-Dataset·GitHub public repository (Ahn et al. [33]).

The dataset we used was introduced by Ahn et al. [33], with full preprocessing details provided within. It includes 799 images of optic disks with no pathology and 295 fundus photographs from patients with papilledema. These photographs were collected at Kim’s Eye Hospital using a non-mydriatic auto fundus camera (AFC-330, Nidek, Gamagōri, Japan). Representative examples of papilledema and normal cases are shown in Figure 1, taken from a publicly available dataset [34].

**Figure 2:**
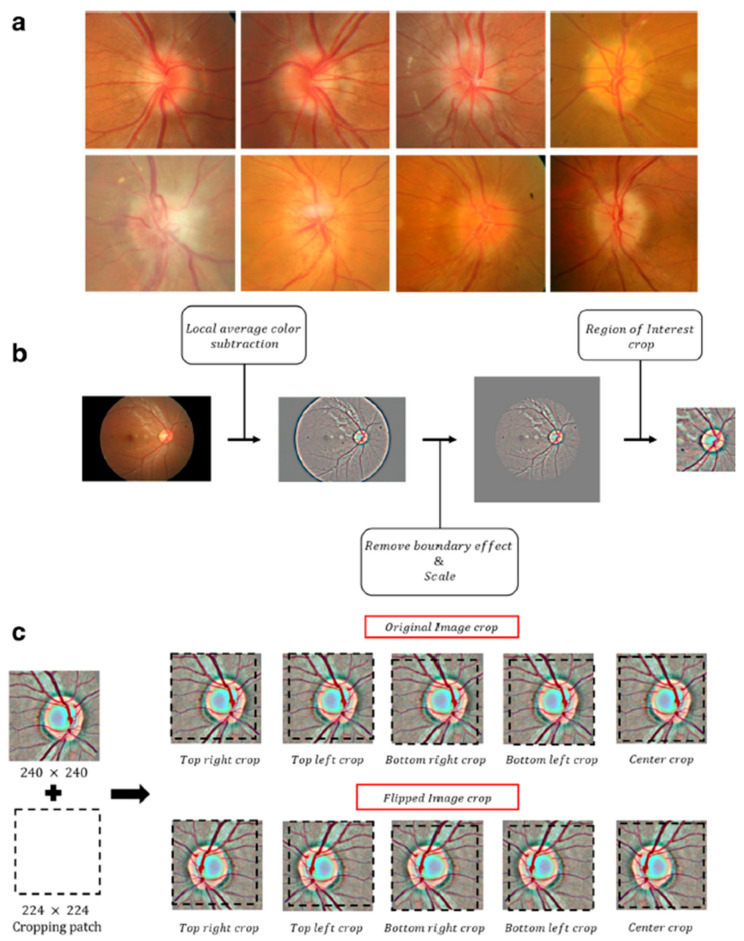
Preprocessing pipeline of the fundus dataset. Preprocessing steps from the publicly available dataset (Ahn et al., 2019 [33], reproduced under CC BY 4.0). (**a**) Representative fundus images (**b**) Preprocessing pipeline including resizing, local average color subtraction with Gaussian filtering, pixel normalization, and cropping around the optic nerve (240 × 240). (**c**) Dataset augmentation scheme, generating five 224 × 224 crops from each image, repeated after horizontal flipping.

**Figure 3:**
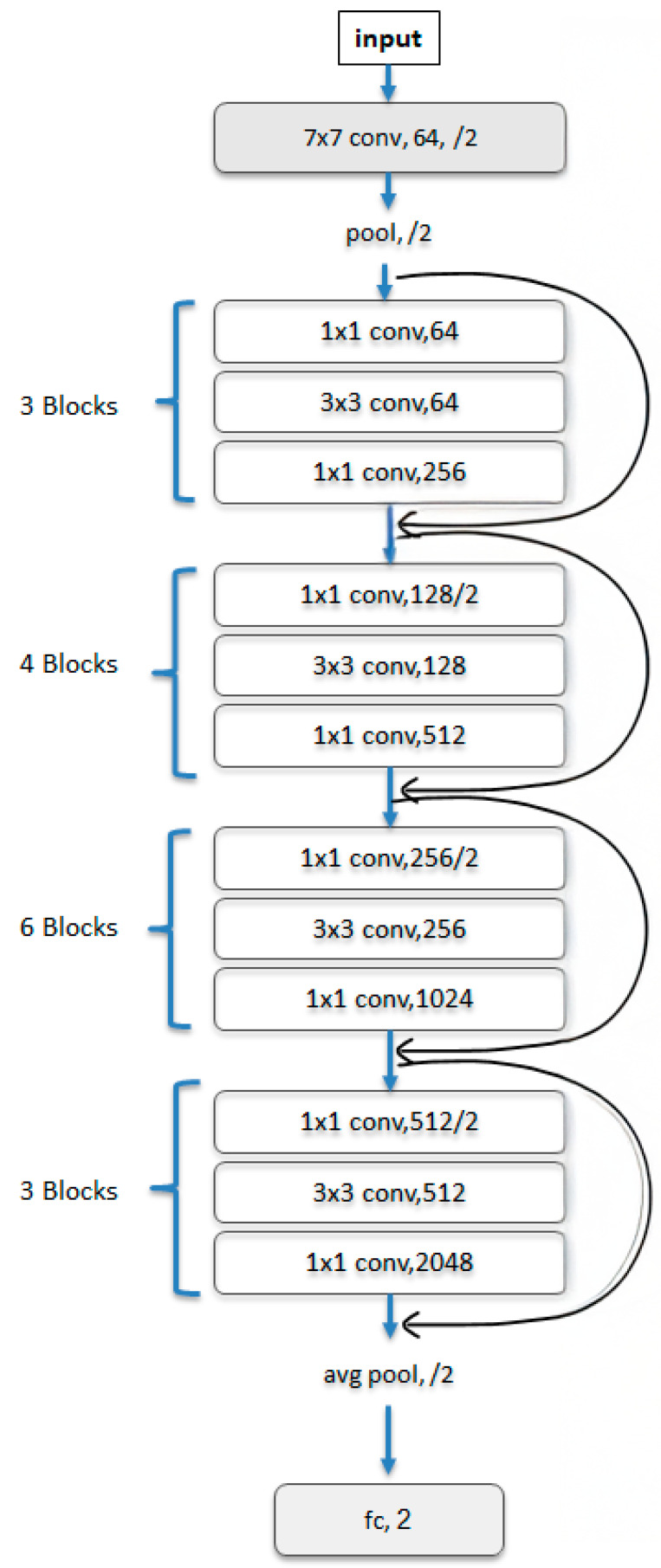
A detailed schematic illustration of the ResNet 50 model. Schematic overview of the ResNet-50 architecture used in this study. The input passes sequentially through multiple weight layers while also being directly added to the output via a shortcut (skip) connection. The final fully connected layer was adapted to output two classes corresponding to our classification task. The convolutional layers consist of kernels ranging from 1 × 1 to 7 × 7, as illustrated in the schematic. The input image size is 224 × 224 pixels. The number of output channels for each stage is indicated within the corresponding block.

This exploratory study was intended to focus exclusively on the binary classification task of distinguishing papilledema from normal fundus images. Images with other optic disk pathologies were excluded from this study. We used the dataset following the preprocessing described in its reference publication [33]. Specifically, images were standardized by resizing to a fixed width of 500 pixels while maintaining their aspect ratio, applying local contrast normalization using Gaussian filtering, normalizing pixel intensities to zero mean and unit variance, and cropping to 240 × 240 pixels centered on the optic nerve. Data augmentation was then performed by generating multiple 224 × 224 crops and horizontal flips for each image. For illustration, Figure 2 (reproduced from the dataset’s reference publication [33] under CC BY 4.0) shows the preprocessing pipeline, including resizing, Gaussian filtering, normalization, and cropping, as well as the dataset’s augmentation scheme. To tackle class imbalance, we defined a test (hold out) set of 99 randomly chosen papilledema images, and 99 randomly chosen normal images, class-balanced to ensure fair evaluation. The validation data used for fine-tuning consisted of 88 patients, randomly selected from the training set. For the classification task, we adopted transfer learning utilizing a powerful CNN (Convolutional Neural Network), ResNet50 (residual learning neural network) [35] model, pretrained on the ImageNet [36] dataset for general image classification. In this architecture, 50 convolutional layers are grouped into residual blocks, where shortcut connections bypass one or more layers to allow stable training of very deep networks. A schematic illustration of ResNet50 architecture with residual blocks and the skip connection is provided in Figure 3. We selected ResNet due to its strong performance in ophthalmic imaging [33,37]. Moreover, its residual connections enable the stable training of deep networks, which has established ResNet as a leading and efficient architecture for image classification [35].

Given the limited size of our dataset, we chose to use discriminative fine-tuning [38], which is particularly suitable for mitigating overfitting by assigning different learning rates to different pretrained model layers. Lower layers are updated conservatively to retain generalizable features, while higher layers are fine-tuned more aggressively to adapt to the task. This targeted adaptation helps prevent the model from fitting to noise while effectively leveraging pretrained knowledge. In addition, we also chose the one-cycle learning rate policy [39], to enhance training efficiency and generalization. This approach allows the model to explore a broader parameter space early in training by gradually increasing the learning rate, followed by a controlled decrease that stabilizes convergence and helps prevent overfitting. The one-cycle policy has been shown to be particularly effective in transferring learning settings involving limited data. Training was performed with a batch size of 64 for 10 epochs using the Adam optimizer with cross-entropy loss. An exponential learning rate schedule (from 1 × 10^−7^ to 10 over 100 iterations) was applied. All experiments were executed on an NVIDIA Tesla T4 GPU (NVIDIA, Santa Clara, CA, USA). We used the publicly available PyTorch implementation version 2.8.0 of ResNet-50 [GitHub—bentrevett/pytorch-image-classification: Tutorials on how to implement a few key architectures for image classification using PyTorch and TorchVision], setting hyperparameters according to the original implementation.

These 198 test images were also evaluated using GPT-4o, a proprietary large language model developed by OpenAI, built on a transformer-based architecture with multimodal input capability (text, images, and audio). The architectural details, including the number of parameters, training dataset composition, and optimization strategy, have not been publicly disclosed. What is publicly known is that GPT-4o integrates vision and language encoders within the transformer framework, enabling direct interpretation of images and generation of descriptive text outputs. In this study, GPT-4o was not trained, tuned, or modified by the investigators; all training procedures described above apply exclusively to the ResNet model. To ensure strict separation of training and evaluation, GPT-4o was only exposed to the independent test set of 198 fundus images, which were not used in ResNet training or validation. Unlike specialized diagnostic AI systems such as ResNet, GPT-4o is not a dedicated ophthalmic diagnostic model; rather, it is a general-purpose model applied here experimentally for papilledema detection. GPT-4o was accessed through its native image input interface (drag-and-drop upload). Each fundus photograph was uploaded individually into a separate chat session, with explicit instructions not to save the images or use them for training. The model was provided only with the raw fundus image, without supplementary text descriptions or patient metadata. While this design ensured a purely image-based evaluation, it may have limited diagnostic performance compared to real-world clinical settings, where metadata such as medical history, medication exposure, and symptoms are considered. To standardize the evaluation, we used a consistent custom GPT configuration (‘Papilledema Detector’), applied to every case. The configuration was as follows: *Papilledema Detector will analyze uploaded fundus images, describe the findings, and determine if there are signs of papilledema. The responses should be clear, concise, and medically accurate, avoiding overly technical jargon. It should focus on describing features related to papilledema, such as optic disk swelling, blurred disk margins, hemorrhages, and exudates. It will not provide medical advice or diagnose conditions, instead suggesting consulting a healthcare professional for a definitive diagnosis. If the image quality is poor or if the uploaded image is not a fundus image, it will ask for clarification. The tone will be clinical, professional, and empathetic.*

Thus, each output included both a descriptive explanation and an explicit categorical judgment. This process follows a reasoning methodology that mirrors the clinical approach, integrating ophthalmologic findings associated with papilledema before reaching a classification decision. For performance analysis, the investigators recorded GPT-4o’s categorical judgment as ‘papilledema’ or ‘normal’. Additionally, to benchmark AI performance against human evaluators, a certified ophthalmologist and an ophthalmology resident independently analyzed the same test images, blinded to GPT-4o’s diagnosis.

### Statistical Analysis

Categorical variables were summarized using descriptive statistics. Interobserver agreement measures were represented using Cohen’s Kappa coefficient. Sensitivity, specificity, positive predictive value, and diagnostic accuracy were calculated for each categorical variable and shown in Table 1. Additionally, a two-sample proportion test was employed to compare the proportions. The data were analyzed using SPSS, version 26.0 for Windows (SPSS, Inc., Chicago, IL, USA). Confusion matrices were constructed for each evaluator (GPT-4, dermatologist, and ophthalmology resident), allowing for the calculation of key performance metrics, including accuracy, sensitivity, specificity, and precision.

## 3. Results

**Figure 4:**
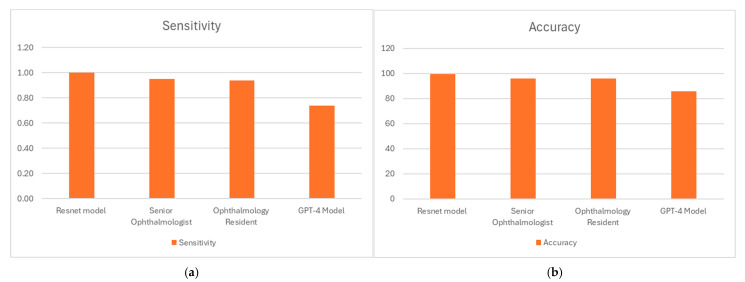

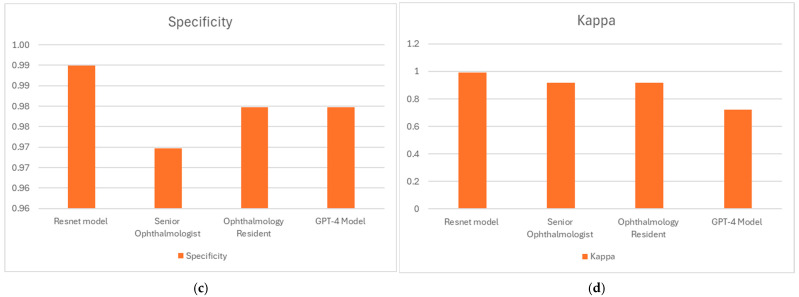
Comparison of the performance of the ResNet model, GPT-4o, a senior ophthalmologist, and an ophthalmology resident on the test dataset. Performance comparison of the ResNet model, GPT-4o, a senior ophthalmologist, and an ophthalmology resident on the test dataset. Shown are (**a**) sensitivity, (**b**) specificity, (**c**) accuracy, and (**d**) Cohen’s kappa.

A total of 198 fundoscopic images comprising the test set were analyzed. The ResNet model, which had rapidly converged during training due to the advantage of pretrained weights, was then evaluated by calculating different performance measures on this test set. The performances of all four evaluators: GPT-4o, the ResNet model, the senior ophthalmologist, and the ophthalmology resident, were estimated on the same test dataset, and the corresponding confusion matrices for each are presented in Table 1. All four evaluators (Figure 4a) demonstrated a high sensitivity for detecting papilledema, ranging from 96.9% (senior ophthalmologist) to 99.0% (ResNet AI model). Specificity (Figure 4b) varied more notably among evaluators, with the highest specificity achieved by the ResNet AI model at 100%, followed by the senior ophthalmologist and ophthalmology resident with specificities of 95.0% and 94.2%, respectively. GPT-4o, which is a multimodal language model rather than a dedicated diagnostic system, exhibited the lowest specificity at 78.9%.

The perfect (100%) positive predictive values (PPV) in the ResNet AI model highlight its reliability in confirming positive diagnoses. These high results were achieved without oversampling or class weighting, underscoring the model’s robustness. Human evaluators had slightly lower PPVs, 94.9% for the senior ophthalmologist and 93.9% for the resident, while GPT-4 showed the lowest PPV of 73.7%, indicating a higher rate of false positives. Negative predictive values (NPV) remained consistently high across all evaluators, with values ranging from 97.0% (senior ophthalmologist) to 99.0% (ResNet AI model), reflecting the high reliability in correctly identifying negative cases.

Overall diagnostic accuracy (Figure 4c) was highest for the ResNet AI model (99.5%), followed by both human evaluators at 96.0%, and lowest for GPT-4 at 86.0%. Cohen’s Kappa values, representing the agreement beyond chance (Figure 4d), were very high for the ResNet AI model (0.99) and similarly high for both human evaluators (0.92), indicating excellent inter-rater reliability among human experts. GPT-4 had a Kappa value of 0.72, signifying substantial agreement.

**Figure 5:**
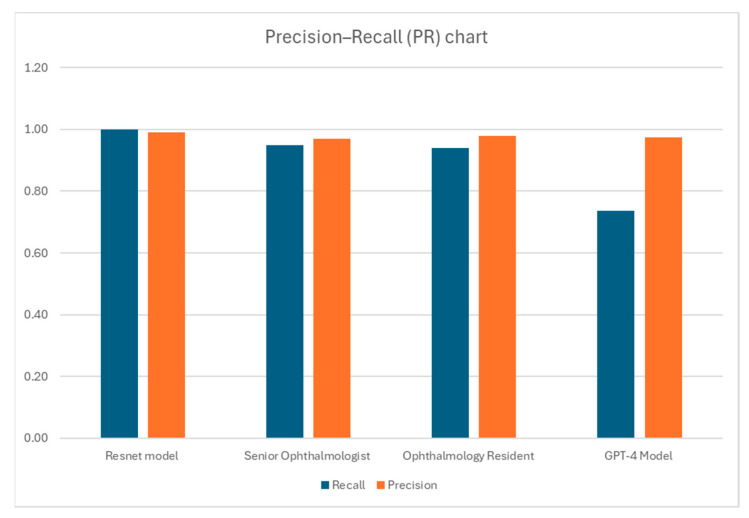
Precision–Recall (PR) analysis of all four evaluators: ResNet model, senior ophthalmologist and ophthalmology resident and the GPT-4 Model. Precision–Recall (PR) analysis of all four evaluators: ResNet model, senior ophthalmologist and ophthalmology resident and the GPT-4 Model.

Precision–Recall (PR) analysis further clarifies clinically relevant aspects of model performance: the reliability of a positive prediction (precision) and the proportion of true positives detected (recall), shown in Figure 5. This visualization highlights that, while the human experts and the ResNet model outperform GPT-4o overall, the LLM achieves high precision but lower recall, reflecting a conservative diagnostic tendency.

## 4. Discussion

The primary objective of this study was to evaluate the ability of GPT-4o, a multimodal large language model, to detect papilledema from fundus photographs. GPT-4o, while showing potential as a generalist multimodal tool, currently underperforms compared to both specialized AI systems and human experts, which aligns with recent studies [25,41,42].

ChatGPT-5 has demonstrated improved performance and greater accuracy over GPT-4o in a variety of tasks, highlighting the rapid progress of these models [43]. Importantly, GPT is not a dedicated diagnostic model; its training was not optimized for ophthalmic image interpretation, and its outputs should therefore be interpreted as exploratory rather than clinically validated. Large language models (LLMs), such as ChatGPT, are increasingly utilized in medical contexts due to their accessibility and public trust. However, their outputs must be interpreted cautiously, especially in high-stakes diagnostic scenarios [44].

The relatively modest specificity and positive predictive value observed with ChatGPT-4o underscore the importance of improving its ability to minimize false positives. While its high precision but lower recall reflects a conservative diagnostic tendency. In the present study, the model achieved an accuracy of 85.9%. Prior research has demonstrated more heterogeneous performance; for instance, Carla et al. [27], where ChatGPT was able to correctly classify the correct diagnosis in 62% of the cases, and Gupta et al. [41], who demonstrated that ChatGPT accurately diagnosed 4 out of 12 fundus images. These findings are consistent with known challenges that large language models face when interpreting complex or ambiguous visual input [23,44], underscoring the need for further refinement before clinical application.

Ahn et al. [33] developed a DLS that distinguished between normal optic disks, papilledema, and other optic disk abnormalities with high accuracy. This laid the foundation for trained models for grading papilledema severity [45]. These systems have demonstrated performance comparable to that of experienced ophthalmologists and emergency physicians, and have maintained robustness across various imaging devices. Fundus photography, particularly non-mydriatic and smartphone-based systems, may effectively enable remote diagnosis [44,46,47].

Our ResNet-based model demonstrated high performance with an accuracy of 99.5%, similar to other recent work trained for papilledema detection, such as Milea et al. (87.5%) [26], Saba et al. (99.9%) [25], and Ahn et al. (98.6%) [33].

The key question is whether a widely available general-purpose model can demonstrate similar potential. From a clinical perspective, dermatological treatments such as isotretinoin and tetracyclines are well-documented causes of intracranial hypertension [4,10], and new-onset headache in patients taking these medications should prompt urgent evaluation for papilledema. Our findings have relevance in such cases occurring in rural and underserved areas as well as in emergency settings, where timely ophthalmologic consultation is often unavailable. Incorporating AI analysis at the point of care could streamline the treatment process and serve as a viable alternative to direct ophthalmoscopy. Such integration, including readily available LLMs, may have lifesaving potential by accelerating diagnosis and referral for patients at risk of permanent future damage to their optic nerves.

Training a model exclusively on images from a single source can result in biased performance; however, in clinical practice, images are derived from diverse settings, highlighting the importance of developing more generalized models. Thus, the disparity in performance between Specialized AI models, including our ResNet model and ChatGPT-4o, likely stems from their respective architectures and training paradigms. Our ResNet model was specifically fine-tuned for papilledema detection using a domain-specific dataset, benefiting from discriminative learning rates and a one-cycle policy, techniques known to improve generalization with limited data [38,39]. ChatGPT, by contrast, represents a general model with limited ophthalmic visual training and is therefore less suited to nuanced fundoscopic interpretation. As it was not specifically trained on the current dataset, its performance can be viewed as independent, in a manner comparable to that of human physicians.

The COVID-19 pandemic has accelerated the adoption of AI-powered telehealth, and continued technological innovation is likely to further support its integration into clinical practice [26]. Such adoption involves economic, ethical, and technological challenges. Cost is a practical consideration. While AI has proven to be cost-effective in screening for diabetic retinopathy [48], its economic impact on the diagnosis of papilledema remains underexplored. Specialized AI models currently demonstrate superior diagnostic performance compared to ChatGPT; however, their implementation may incur higher costs [49,50]. Although ChatGPT currently falls short of these specialized tools in accuracy, its accessibility, lower cost, and ongoing development suggest it could become a valuable tool for papilledema screening in the future. Continued evaluation of newer ChatGPT versions is necessary to assess their evolving diagnostic capabilities and limitations.

Ethical challenges include concerns around data privacy, algorithmic bias, and clinical accountability [20,51]. Technological challenges include overcoming low-quality fundus images outside the ophthalmology clinics, for instance, when using a mobile fundus camera, which impacts AI diagnostic accuracy [46,52,53]. Doing so requires modifications for improved imaging quality, including pupil dilation, lighting adjustments, lens and camera positioning, Video Capture Mode, and app-based controls [46]. Another technological challenge involves equipment availability, including the lack of dedicated fundus cameras and reliance on mobile or smartphone-based solutions. Operator training outside ophthalmology clinics remains limited, as acquiring diagnostic-grade fundus photographs requires specialized skills. Studies have shown that image quality often degrades when captured by inexperienced users or by patients themselves, resulting in poor focus [46]. To integrate AI-based tools into non-ophthalmology settings, it will be necessary to equip emergency rooms with dedicated fundus cameras and to establish training programs for reliable image acquisition. All such tools should undergo rigorous assessment before implementation.

In the future, patients may be able to capture their own fundus images using smartphone-based fundus photography, with AI algorithms embedded in electronic medical records enabling real-time risk stratification and referral [54,55].

Looking ahead, human-in-the-loop systems [56], where AI supports screening and triage while final decisions remain with clinicians, could enhance safety and efficiency, particularly in rural or resource-limited settings. The potential impact of AI misdiagnoses on clinical decision-making should be acknowledged. Misclassification of papilledema as normal could delay referral and risk vision or life-threatening consequences, whereas false positives could lead to unnecessary anxiety, referrals, and investigations. Moreover, there is a risk of automation bias, whereby clinicians may be unduly influenced by AI-generated outputs, potentially resulting in false reassurance or over-investigation. These considerations further underscore the importance of validating AI models, transparently reporting their limitations, and incorporating them into carefully designed human-in-the-loop workflows that safeguard independent clinician judgment.

## 5. Limitations

Our study has several limitations. First, the performance gap observed between the models may partly reflect differences in training scope. GPT-4o is a general-purpose model not trained explicitly for our purpose. In contrast, the ResNet model was trained and validated using a single-institution dataset obtained from a single non-mydriatic fundus camera model. Using larger, multi-center datasets collected with different imaging devices, including smartphone-based or handheld fundus cameras, and incorporating such images into training could improve robustness and result in the following dual effects: On the one hand, this would enhance clinical applicability across diverse patient populations and imaging settings; on the other hand, it might also alter ResNet’s performance and potentially reduce the observed performance differences between the models. Moreover, the standardized preprocessing applied to images may not fully reflect the real-world variability in imaging quality and modalities encountered in routine clinical practice, which could potentially affect diagnostic performance in practical settings. Since variability in device optics and image quality may impact diagnostic accuracy, future studies should investigate the robustness of our approach across multiple platforms, devices, and operators.

Second, we did not incorporate patient-level clinical metadata such as medication history, symptoms, or neurological findings, which typically play a significant role in real-world diagnostic decision-making. Future studies that incorporate detailed anamnestic data and health records could also enable the evaluation of other optic disk pathologies, including subgroups of pseudo-papilledema. That said, when attempting to distinguish between papilledema and a normal fundus, the absence of clinical information can be an advantage in some cases. Anamnestic input may introduce a degree of bias, whereas the algorithmic approach could offer a more objective and potentially less confounded assessment [23,42].

Lastly, the exact training datasets and methods used for GPT-4o are unknown, creating uncertainty regarding the ophthalmologic content it was exposed to during training and limiting transparency in interpreting its diagnostic decisions. Future work can integrate explainable AI models to complement this study, elevating the interpretability of the model’s diagnostic outputs.

## 6. Conclusions

This study explored the capability of GPT-4o, a large language model with multimodal input, to detect papilledema from fundus photographs. GPT-4o achieved moderate diagnostic accuracy and substantial agreement with the ground truth, but it underperformed compared to both a domain-specific ResNet model and human ophthalmologists. These findings underscore the distinction between generalist large language models and specialized diagnostic AI: while GPT-4o is not optimized for ophthalmic imaging, its accessibility, adaptability, and rapid evolution highlight its potential as a future adjunct in clinical screening, particularly in underserved settings. Broader validation across diverse datasets and imaging devices, as well as integration of clinical metadata, will enhance its potential.

## Figures and Tables

**Table 1:** (a–d) Confusion Matrices Comparing Evaluator Performance Against Ground Truth Labels for Papilledema Detection. (a) Confusion Matrix for ResNet AI Model in Detecting Papilledema Compared to Ground Truth. (b) Confusion Matrix for Senior Ophthalmologists in Detecting Papilledema Compared to Ground Truth. (c) Confusion Matrix for Ophthalmology Resident in Detecting Papilledema Compared to Ground Truth. (d) Confusion Matrix for GPT-4 Model in Detecting Papilledema Compared to Ground Truth.

(**a**) ResNet AI Model			
	Resnet model P *	Resnet model N **	**Total**
Labeled P	99	0	99
Labeled N	1	98	99
Total	100	98	198
Sensitivity	1.0		
Specificity	0.99		
Accuracy (%)	99.5		
Cohen’s Kappa	0.99		
(**b**) Senior Ophthalmologists			
	Senior Ophthalmologist P *	Senior Ophthalmologist N **	**Total**
Labeled P	94	5	99
Labeled N	3	96	99
Total	97	101	198
Sensitivity	0.95		
Specificity	0.97		
Accuracy (%)	95.96		
Cohen’s Kappa	0.9192		
(**c**) Ophthalmology Resident			
	Ophthalmology Resident P *	Ophthalmology Resident N **	**Total**
Labeled P	93	6	99
Labeled N	2	97	99
Total	95	103	198
Sensitivity	0.94		
Specificity	0.98		
Accuracy (%)	95.96		
Cohen’s Kappa	0.92		
(**d**) GPT-4 Model			
	GPT-4o model P *	GPT-4o model N **	**Total**
Labeled P	73	26	99
Labeled N	2	97	99
Total	75	123	198
Sensitivity	0.74		
Specificity	0.98		
Accuracy (%)	85.86		
Cohen’s Kappa	0.72		

* P = Papilledema; ** N = Normal; Kappa Agreement Scale [40]; 0.01–0.20: Slight agreement. 0.21–0.40: Fair agreement. 0.41–0.60: Moderate agreement. 0.61–0.80: Substantial agreement. 0.81–1.00: Almost perfect (or strong) agreement.

## Data Availability

The data supporting the findings of this study are available from the corresponding author upon request, owing to ethical restrictions.
